# Towards Aldehydomics: Untargeted Trapping and Analysis of Reactive Diet-Related Carbonyl Compounds Formed in the Intestinal Lumen

**DOI:** 10.3390/antiox10081261

**Published:** 2021-08-06

**Authors:** Sylvie Chevolleau, Maria-Helena Noguer-Meireles, Loïc Mervant, Jean-François Martin, Isabelle Jouanin, Fabrice Pierre, Nathalie Naud, Françoise Guéraud, Laurent Debrauwer

**Affiliations:** 1 Toxalim, Research Centre in Food Toxicology, Université de Toulouse, INRAE, ENVT, INP-Purpan, UPS, CEDEX 3, 31027 Toulouse, France; mariahelena.noguer@gmail.com (M.-H.N.-M.); loic.mervant@inrae.fr (L.M.); jean-francois.martin@inrae.fr (J.-F.M.); isabelle.jouanin@inrae.fr (I.J.); fabrice.pierre@inrae.fr (F.P.); nathalie.naud.31@inrae.fr (N.N.); francoise.gueraud@inrae.fr (F.G.); laurent.debrauwer@inrae.fr (L.D.); 2 Metatoul-AXIOM Platform, National Infrastructure for Metabolomics and Fluxomics, MetaboHUB, CEDEX 3, 31027 Toulouse, France

**Keywords:** metabolomic, untargeted trapping, aldehydes, 4-hydroxy-nonenal, 4-hydroxy-hexenal, malondialdehyde, HPLC–HRMS, isotopic labelling, fecal water, lipid peroxidation

## Abstract

Lipid peroxidation and subsequent formation of toxic aldehydes, such as 4-hydroxynonenal, is known to be involved in numerous pathophysiological processes, possibly including the development of colorectal cancer. This work aimed at the development of an untargeted approach using high-performance liquid chromatography coupled with high-resolution mass spectrometry (HPLC–HRMS) for tracking aldehydes in both suspect screening and untargeted methods in fecal water, representing the aqueous environment of colon epithelial cells. This original approach is based on the introduction of a characteristic isotopic labeling by selective derivatization of the carbonyl function using a brominated reagent. Following a metabolomics workflow, the developed methodology was applied to the characterization of aldehyde compounds formed by lipid peroxidation in rats fed two different diets differentially prone to lipoperoxidation. Derivatized aldehydes were first selectively detected on the basis of their isotopic pattern, then annotated and finally identified by tandem mass spectrometry. This original approach allowed us to evidence the occurrence of expected aldehydes according to their fatty acid precursors in the diet, and to characterize other aldehydes differentiating the different diets.

## 1. Introduction

Recent epidemiological studies have shown that diets rich in red or processed meat are associated with an elevated risk of colorectal cancer (CRC), whereas those rich in fish and white meat are not [1,2,3]. As of its 2007 report [4], and more recently in its 2018 Continuous Update Project [5], the World Cancer Research Fund (WCRF) panel recommended limiting red meat and avoiding processed meat consumption to limit the risk of colorectal cancer. One of the major hypotheses of this association involves dietary heme iron, the concentration of which is much higher in red meat than in white meat, and which could promote CRC by inducing or enhancing luminal dietary lipid oxidation [6,7].

The oxidation of dietary polyunsaturated fatty acids (PUFAs) is a non-enzymatic free-radical-mediated peroxidation induced by, for instance, ions of metals such as iron. It occurs in three steps: (1) the initiation step, in which the abstraction of a hydrogen atom from the fatty acid chain gives rise to a lipid radical; (2) the propagation step, in which the lipid radical reacts with dioxygen to produce a lipoperoxyl radical that, in turn, reacts with another lipid chain; and (3) the termination step, in which radical species combine to produce non-propagating species. All of these lipid intermediates can undergo β-scission and give rise to the reactive formation of secondary lipid oxidation products—such as aldehydes, and in particular 4-hydroxy-alk-2,3-enals—which display electrophilic features, making them reactive towards nucleophilic biomolecules—especially proteins, but also lipids and DNA [8,9]. Indeed, several secondary lipoperoxidation products, including these aldehydes, can be formed not only in the diet (particularly in heme-iron- and polyunsaturated-fat-rich foods), but also during digestion in the intestinal lumen, with pro-oxidative conditions favoring their production [10].

Aldehydes, and in particular hydroxyl-alkenals such as (2E)-4-hydroxy-non-2-enal (HNE) and (2E)-4-hydroxy-hex-2-enal (HHE)—formed from ω6- (e.g., linoleic and arachidonic acid) and ω3- (e.g., α-linolenic acid) PUFAs, respectively—are known to be cytotoxic and genotoxic. When present in the bioavailable faction of feces (fecal water), such aldehydes may play a promoting role in the development of CRC, by selecting precancerous colon epithelial cells [6,11].

The detection and quantification of such species in the intestinal lumen is thus of particular interest. Due to their volatility (especially for short-chain aldehydes) and their reactive properties (e.g., binding, decomposition), the analysis of aldehydes remains challenging. Several analytical approaches have been developed for the sensitive detection and characterization of aldehydes as lipid peroxidation products. As a general feature, due to their physicochemical properties, a derivatization step is required for their gas chromatography (GC) analysis. This is also the case for their liquid chromatography (LC) analysis, since they display poor UV or fluorometric responses as well as poor MS ionization yields, and in most cases they first need to be stabilized. For aldehyde derivatization, several reactants have been used, mainly involving hydrazine and hydroxylamine chemistry. The most popular methods use pentafluorobenzyl hydroxylamine (PFBHA) for GC analysis [12] and dinitrophenylhydrazine (DNPH) for LC analysis [13]. However, the selectivity of the DNPH reagent towards carbonyl compounds has been questioned [14].

Mass spectrometry coupled with GC or LC is now the most popular technique for the selective and sensitive screening and quantification of molecules in various matrices, particularly with the use of triple-quadrupole mass spectrometers operating in the multiple reaction monitoring (MRM) mode. This technique currently provides the most sensitive detection method when applied to known targeted compounds, and has been extensively used for the analysis of several oxidation products—such as aldehydes and ketones—in various matrices [15,16,17,18,19]. However, depending on the chain length and the unsaturation degree and position of the fatty acids, lipid peroxidation is known to give rise to numerous and various carbonyl compounds. Although specific to their precursor fatty acids, these compounds are still not fully identified, and current targeted detection methods may not provide complete coverage of aldehyde formation as a result of lipid peroxidation in the intestinal lumen. In order to get the most complete coverage, the use of full-scan untargeted or semi-targeted metabolomics approaches performed in high-resolution mass spectrometry (HRMS) represents one of the most promising alternative solutions. Although the HRMS acquisition mode remains less sensitive than the MRM mode, time-of-flight (TOF) or Orbitrap mass analyzers are now more and more frequently used for screening and biomonitoring applications [20,21,22].

In the context of aldehyde profiling, the derivatization strategy should aim at (1) incorporating chemical groups that allow stabilization of the aldehyde function and enhancement of the ionization yields in order to improve sensitivity, and (2) incorporating stable isotopes to work on derivatives displaying characteristic isotopic patterns, thus allowing the untargeted but specific screening of derivatives by means of signal filtering. Benzenaminium halides proposed by Eggink et al. fulfill the first criterion, and can even fulfill both when considering the corresponding isotopically labelled reagents introduced either via a halogen atom or a mixture of “normal” and “heavy” reagents [23,24,25,26]. Hydrazinotriazines were also proposed as derivatization reagents for fatty aldehydes [27], and could also be used for profiling by monitoring specific neutral losses using deuterated hydrazinotriazines. More recently, another method has been proposed for isotope-coded derivatization of aldehydes using ^14^N/^15^N-ammonium acetate and 9,10-phenanthrene quinone [28].

In this work, we present the development of an “aldehydomics” workflow based on the untargeted aldehyde characterization method in fecal water, using aldehyde stabilization by quantitative in situ derivatization under mild conditions before LC–HRMS analysis. In this approach, we selectively labeled aldehydes with a bromine atom to take advantage of the specific detection of its characteristic ^79^Br/^81^Br isotopic pattern, as already used for pharmaceutical compounds or chemical contaminants [29,30]. For this, we propose here the use of the brominated derivatizing reagent 1-((ammoniooxy)methyl)-2-bromobenzene chloride (BBHA), which has already proven to be well suited for the targeted analysis of HNE and HHE [31]. To validate our proof of concept, this approach was then applied to the tracking of free aldehydes in fecal waters prepared from rats fed two diets containing saturated (namely, coconut oil (COHE)) or unsaturated (namely, linseed oil (LIHE)) fatty acids with added pro-oxidant heme iron. Aldehydes whose formation could be expected according to the diet could be characterized, and several other brominated signals corresponding to potentially unknown aldehydes were also evidenced.

## 2. Materials and Methods

### 2.1. Chemicals

Methanol (HPLC grade/Optima LC/MS grade) and acetonitrile (Optima LC/MS grade) were purchased from Fisher (Illkirch, France), while formic acid was purchased from Sigma-Aldrich (Saint-Quentin-Fallavier, France). Ultrapure water was obtained using a Milli-Q system (Millipore, Saint-Quentin-en-Yvelines, France).

4-(2-((4-Bromophenethyl)dimethylammonio)ethoxy) benzenaminium dibromide (APEBA) was prepared and used as described by Eggink et al. [25]. 1-((Ammoniooxy)methyl)-2-bromobenzene chloride (BBHA) was purchased from Interchim (Montluçon, France). Piperazine-N,N′-bis(2-ethanesulfonic acid) (PIPES) and trifluoroacetic acid (TFA) were purchased from Acros Organics (Geel, Belgium).

Standard HHE, HNE, 4-hydroxynonanal (OHN), and ω-oxo-carboxylic acids (5-oxo-pentanoic acid (5-OPA), 6-oxo-hexanoic acid (6-OHA), 7-oxo-heptanoic acid (7-OHA), 8-oxo-octanoic acid (8-OOA), 9-oxo-nonanoic acid (9-ONA), 10-oxo-decanoic acid (10-ODA)) were synthesized in house according to previously published methods [32,33]. All other standard aldehydes and other standard compounds were purchased from Sigma-Aldrich (Saint-Quentin-Fallavier, France), except for 9-oxo-10,12-octadecadienoic acid (9-OxoODE), 9-Oxo-10,12,15-octadecatrienoic acid (9-OxoOTrE), and α-linolenic and eicosapentaenoic acid which were purchased from Bertin Bioreagent (Montigny-le-Bretonneux, France).

### 2.2. Animal Experiment and Fecal Water Preparation

Animal experiments were carried out with seven-week-old male Fisher 344 rats (6 rats/group) purchased from Charles River Laboratories (Les Oncins, France). Animal care was in accordance with our local ethics committee (number TOXCOM/006/FG). Rats were housed in individual plastic metabolic cages equipped for fecal collection. Their diets were based on a modified AIN-76 diet prepared and formulated in a powdered form by UPAE (Jouy-en-Josas, France). Each diet was formulated with 5% oil—either hydrogenated coconut oil (COHE, MP Biomedicals, Solon, OH, USA) or raw linseed oil (LIHE, MP Biomedicals, Solon, OH, USA)—and 0.94 g of hemin (Sigma-Aldrich, Saint-Quentin-Fallavier, France) per kg of diet. To minimize diet lipid peroxidation during storage, oils and hemin were added to the powdered diet just before the start of the experiments. After preparation, each diet was aliquoted into individual portions for each experimental day, and stored at −20 °C until use. Rats were fed their respective experimental diet ad libitum, at the end of each day, just before their active period. Diet consumption was recorded every two days. Feces were collected over 24 h on day 13 of the experimental diets. Fecal water was prepared by adding 0.5 g of feces to 1 mL of distilled water and 50 µL of 0.45 M BHT in ethanol (2,6-Di-tert-butyl-4-methyl-phenol). The resulting samples were then processed using FastPrep technology and then centrifuged at 3200× *g* for 20 min. After centrifugation, supernatants were sampled and immediately submitted to derivatization for aldehyde stabilization.

### 2.3. Sample Treatment

Derivatization with APEBA was carried out as described by Eggink et al. [25]. In brief, fecal water was derivatized with APEBA at pH 5.7 (NH_4_OAc 150 mM), in the presence of NaBH_3_CN, for 3 h at 8 °C. Aldehyde-APEBA derivatives were then extracted with dichloromethane, evaporated to dryness, and reconstituted in a CH_3_OH–H_2_O (1:1) mixture for LC–HRMS analysis.

For BBHA derivatization, reaction conditions were investigated in a previous work [33] on several aldehydes and ketones. Special attention was given to testing the reactivity of BBHA towards carbonyl compounds at low temperatures and a slightly acidic pH to preserve the integrity of the aldehyde function. According to these adjusted conditions, fecal water samples (100 µL) were added to 60 µL of PIPES buffer (0.1 M, pH 6.5) and derivatized with 120 µL of BBHA (50 nmol/µL) in PIPES buffer. The samples were stirred at 6–8 °C for one hour.

**Figure 1:**
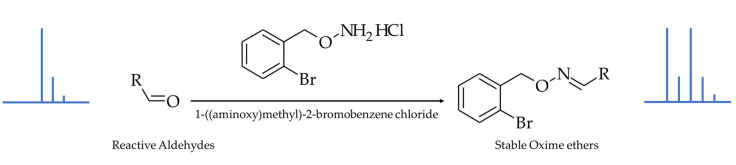
Derivatization reaction and chemical structures of the resulting oxime ethers, showing the introduction of the characteristic brominated isotopic pattern.

The derivatization reaction and the chemical structures of the BBHA derivatives of HNE and HHE—the two most representative hydroxy-alkenals—are shown in Figure 1.

SPE purification of the derivatized extracts was conducted on a Visiprep SPE Vacuum Manifold (Supelco, Saint-Quentin-Fallavier, France), using Agilent Bond Elut C18 (100 mg, 1 mL) cartridges. The sorbent was conditioned with 1 mL of CH_3_OH, then 1 mL of water. The derivatized samples were vortexed and then deposited on the SPE cartridge. Washing was first performed with 1 mL of PIPES (rinsing the container and depositing on the cartridge), and then with 0.05% TFA/CH_3_OH (3/2). The cartridge was then dried under vacuum (1 min), eluted with 400 μL of CH_3_OH, and collected in glass tubes. Finally, the volume was adjusted to exactly 400 μL with CH_3_OH, and the extracts were stored at −20 °C until analysis.

### 2.4. Liquid Chromatography–Mass Spectrometry

Sample extracts were analyzed via high-performance liquid chromatography coupled with high-resolution mass spectrometry (HPLC-HRMS). The HPLC system consisted of an Ultimate 3000 RS pump fitted with the Ultimate 3000 autosampler (Dionex, Thermo Scientific, Les Ulis, France). The chromatographic pump was operated at a flow rate of 0.200 mL/min with the following elution gradient program—0 min: 0% of B; from 3 min to 15 min: 100% of B; and from 15 to 25 min: 100% of B. Mobile phases were composed of (A) H_2_O/CH_3_CN/HCOOH 95/5/0.1 (*v*/*v*/*v*) and (B) CH_3_CN/H_2_O/HCOOH 95/5/0.1 (*v*/*v*/*v*). Five microliters of of sample was injected into a Kinetex C18 (150 × 2.1 mm, 2.6 μm) column (Phenomenex, Le Pecq, France) maintained at 40 °C. Detection was achieved on an LTQ-Orbitrap XL hybrid mass spectrometer (Thermo Scientific, Les Ulis, France) equipped with an electrospray ionization source used in the positive mode. Ionization parameters were set at +4.5 kV for the spray voltage, 35 arbitrary units (au) for the sheath gas flow rate (N_2_), 5 au for the auxiliary gas flow rate (N_2_), and 300 °C for the capillary temperature. High-resolution mass spectra were acquired at a resolution power of 30,000 from *m*/*z* 80 to 800 in centroid mode. Identifications were carried out via tandem mass spectrometry experiments (MS/MS) using the ion trap mass analyzer of the LTQ-Orbitrap mass spectrometer (NCE = 25%). Solutions of synthesized standard HHE-BBHA and HNE-BBHA at different concentrations were used to characterize the method in terms of linearity of response, repeatability, and sensitivity.

### 2.5. Data Processing and Statistical Treatment

Features corresponding to brominated compounds were extracted from raw data using XCMS software. The centWave algorithm was used with a scan-to-scan deviation value set to 10 ppm. The chromatographic peak width was set between 10 and 40 s. For the alignment we used an *m*/*z* range of 0.01 (mzwid) and a retention window of 20 s (bandwidth). Based on the HRMS signal of the exact mass of each [M+H]^+^ ion, features were extracted according to a mass measurement error of ±5 ppm, and to the occurrence of two signals of equivalent intensities (ratio of intensities between 0.667 and 1.333), with ΔM = 1.998 (±0.001) corresponding to the mass difference between the two bromine isotopes [34]. Following their detection, structural characterization of potential aldehydes was carried out by processing MS^n^ spectra using Xcalibur Qual Browser (Thermo Scientific, Les Ulis, France).

In order to target aldehydes of interest, we used statistical treatment to discover a list of features differentiating the studied diets. Both multivariate unsupervised principal component analysis (PCA) and supervised partial least squares discriminant analysis (PLS-DA) were used. PLS-DA aims to find the most discriminating features using the variable importance in projection (VIP) criterion. A VIP greater than 1 gives a first list of potential discriminant features. A univariate, non-parametric Kruskal–Wallis test was eventually carried out, and features with a Kruskal–Wallis *p*-value lower than 0.05 define the list of potential aldehyde ions.

## 3. Results and Discussion

### 3.1. Set-Up of the Derivatization Method

**Figure 2:**
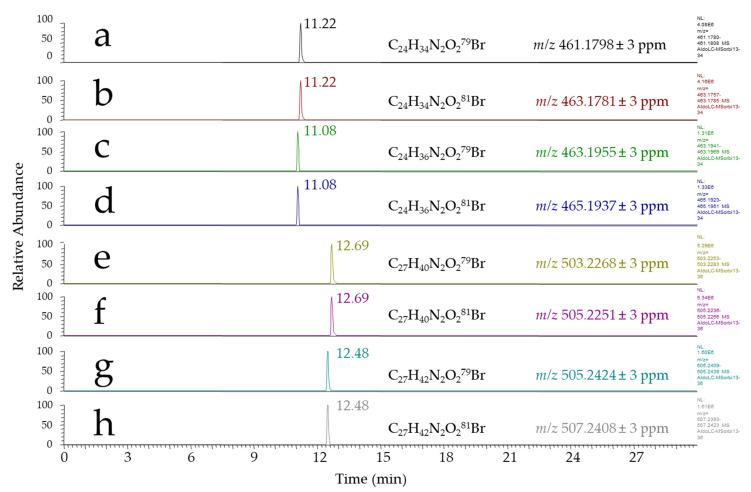
LC–HRMS-extracted chromatograms: (**a**,**b**) ion chromatograms of the ^79^Br and ^81^Br isotopomers of HHE-APEBA; (**e**,**f**) ion chromatograms of the ^79^Br and ^81^Br isotopomers of HNE-APEBA; and (**c**,**d**,**g**,**h**) ion chromatograms of their corresponding reduction byproducts.

Based on the work of Eggink et al., we first investigated 4-(2-((4-bromophenethyl)dimethylammonio)ethoxy) benzenaminium dibromide (APEBA) for derivatizing aldehydes in fecal water. This reactant was designed for a selective derivatization of aldehydes under reductive conditions using NaBH_3_CN, and has been successfully applied to the detection of saturated aldehydes (i.e., pentanal, hexanal, heptanal, octanal, nonanal, and decanal) [25]. Using APEBA, the expected responses for saturated aldehydes were observed at nominal *m*/*z* of, for example, 461/463, 475/477, and 489/491 for heptanal, octanal, and nonanal, respectively, in agreement with previously reported results. Indeed, since those aldehydes represent lipid peroxidation products of low toxicological concern, we focused our attention on more reactive species, among which HHE and HNE were considered as reference compounds for the setup of our method. Thus, APEBA derivatives of standard HHE and HNE were synthesized and analyzed by LC–HRMS. Figure 2 shows the chromatograms obtained from the injection of standard HHE and HNE derivatized using APEBA as described by Eggink et al. [25]. Along with the expected HHE-APEBA and HNE-APEBA derivatives observed at *m*/*z* 461.1798/463.1781 and 503.2268/505.2251, respectively (Figure 2a,b,e,f), the occurrence of additional signals corresponding to brominated compounds was also noticed, most likely arising from aldehyde-APEBA derivatives, at *m*/*z* 463.1955/465.1937 and 505.2424/507.2408, respectively (Figure 2c,d,g,h). These *m*/*z* ratios corresponded to the chemical formulae C_24_H_36_N_2_O_2_Br and C_27_H_42_N_2_O_2_Br, i.e., two additional hydrogen atoms with respect to HHE and HNE derivatives, respectively. These signals represented ~10–20% of the signals measured for HHE-APEBA and HNE-APEBA, and very likely resulted from the partial reduction of the unsaturated HNE or HHE aldehydes—although the chemical explanation for this reduction remains unclear, since NaBH_3_CN is expected to selectively reduce the imine function formed by the reaction of the aldehyde with APEBA.

These kinds of byproducts were also observed when derivatizing standard 4-oxo-hex-2-enal (OHE) and 4-oxo-non-2-enal (ONE), for which signals corresponding to HHE-APEBA and HNE-APEBA were present (data not shown), suggesting that the 4-oxo group was reduced to 4-hydroxyl. Note that unsaturated aldehydes such as 2-heptenal and 2-nonenal were also tested under the same conditions, and produced APEBA derivatives of both heptenal and heptanal in the first case, and both nonenal and nonanal in the second case (data not shown). However, to the best of our knowledge, the APEBA derivatization methodology has only been applied to linear saturated aldehydes or aldehydes that are not prone to reductive processes. This is also the case in the work recently published by Yu et al. [26], using APC—a reagent similar to APEBA previously developed by Eggink et al. [25]. Among other aldehydes, HNE and HHE were also analyzed as APC derivatives by these authors [24], but the search for potential reduction byproducts of these two hydroxy-alkenals is not mentioned.

Since this feature could yield unexpected byproducts and lead to false positive results for several compounds of interest due to an artefactual reduction process, several other reaction conditions were assessed to avoid the formation of such reduction byproducts using APEBA, but all of our trials failed. Therefore, the use of a reagent allowing the selective derivatization of aldehydes under non-reductive, mild, cold, and neutral conditions had to be considered. We selected a simple, commercially available reagent—namely, 1-((ammoniooxy)methyl)-2-bromobenzene chloride (BBHA), which reacts with free carbonyl compounds [33] in one step, with no co-reagent, following a very simple and quick protocol, under mild conditions (no degradation due to chemical conditions) and at low temperature (important because of the volatility of some aldehydes). Similarly to the case of APEBA, high-resolution mass spectrometric analysis allowed a sensitive and selective detection of derivatized aldehydes on the basis of the ^79^Br and ^81^Br isotopomers of the BBHA derivatives (isotopic pattern, exact mass, mass defect).

Although method validation was not our main scope, standard HHE- and HNE-BBHA derivatives were used to evaluate the performances of the method in terms of linearity, repeatability, and sensitivity of detection. For that, standard mixtures of HHE-BBHA and HNE-BBHA (10, 20, 50, 100, 250, and 500 pg/µL) were analyzed. The retention time RSD was found to be <1% for both derivatives (*n* = 3). The RSD values calculated from the peak areas of both derivatives were found to be <15% over the whole concentration range (*n* = 3 for each concentration point). The LOD was reached for 10–15 pg (30–40 fmol) injected into the column based on HHE and HNE, which is around 5–10 times higher than the LOD obtained by targeted analysis of these two compounds using the same derivatization reagent. Compared to other published aldehyde screening methods, these values are in the same range as the LOD reported by Eggink et al. [24], and slightly higher than that reported by Yu et al. [26] with the APC reagent, likely due to the fact that (1) BBHA is not a charged reagent as compared to APC and APEBA, and (2) the response was split over the two bromine isotopomers of the BBHA derivatives as compared to APC. The linearity of the response was checked over the 10–500 pg/µL range, and gave R^2^ values of 0.994 and 0.997 for HHE-BBHA and HNE-BBHA, respectively, enabling the use of this method at least in a semi-quantitative way.

The selectivity of the derivatization method has been assessed with several carbonyl compounds, including aldehydes and ketones, as well as carbohydrates. Carbohydrates that could be present in the intestinal lumen can also be derivatized with BBHA when occurring in their reductive form. As expected, those species bearing several hydroxyl groups yielded characteristic MS/MS spectra with intense [MH−H_2_O]^+^ ions, weak signals at *m*/*z* 168.9647, and a common fragment ion at *m*/*z* 255.9972 (data not shown). Conversely, no BBHA derivative was formed from any standard fatty acid tested in the derivatization medium.

### 3.2. MS/MS Fragmentation of Aldehyde BBHA Derivatives

The synthesis and spectral characterization (^1^H NMR, exact molecular mass) of 11 BBHA derivatives of standard carbonyl compounds (9 aldehydes and 2 ketones) has been published elsewhere [33]. To enable efficient suspect screening data mining from high-resolution full-scan acquisitions, other compounds of interest were further selected and synthesized, and MS/MS spectra were acquired for a total of 16 derivatized carbonyl compounds. Standard derivatized aldehydes are listed in Table 1 together with their molecular formulae, calculated exact mass, and the major fragment ions observed in MS/MS experiments.

The MS/MS spectra of all derivatives display the *m*/*z* 168.9646 fragment ion (C_7_H_6_Br) as base peak, resulting from the cleavage of the C–O bond of the oxime ether, leading to the bromo-benzyl carbocation. This diagnostic fragment ion could be used as tracer for untargeted unknown searching using, for example, all-ion (DIA) MS/MS strategies. Interestingly, hydroxy-alkenals such as HHE and HNE display an intense fragment ion at *m*/*z* 224.0069, which may be considered characteristic of hydroxy-alkenals. This particular *m*/*z* ratio fits the chemical formula C_10_H_11_NBr. The hydroperoxide homolog of HNE (HPNE) displays the same type of fragment ion, but at *m*/*z* 240.0019 (C_10_H_11_NOBr)—i.e., shifted by 15.995 AMU—corresponding to one oxygen atom, suggesting that these compounds behave in the same way towards collisional activation. The formation of this fragment ion must involve a rearrangement in which the oxygen atom of the ether oxime function is eliminated. However, this fragmentation mechanism was not investigated further. Furthermore, the MS/MS spectrum of HPNE also displays a fragment ion at *m*/*z* 322.0807, which fits the elimination of H_2_O_2_ from the MH^+^ parent ion.

Two 4-oxo alkenals were also analyzed—namely, OHE and ONE. As expected, the *m*/*z* 169 ion was also the base peak for both compounds. As reported in Table 1, both compounds displayed a loss of CO as well as an *m*/*z* 221.9913 fragment ion in agreement with the chemical formula C_10_H_9_NBr, suggesting that 4-oxo alkenals undergo the same rearrangement as their 4-hydroxy analogs. However, in the case of 4-oxo alkenals such as OHE and ONE, a fragment ion was also observed at *m*/*z* 240.0020, corresponding to C_10_H_11_ONBr, which could be attributed to an α-cleavage relative to the CO group.

The saturated hydroxylated aldehyde 4-hydroxynonanal (OHN), as well as ω-oxo carboxylic acids (5-OPA, 6-OHA, 7-OHA, 8-OOA, 9-ONA, and 10-ODA), all display an [MH-H_2_O]^+^ ion as one of the most intense fragments. These compounds with a non-functionalized carbon chain then undergo a breakage of the N–O bond from the ether-oxime function of their BBHA derivative, giving rise to the *m*/*z* 96.0044, 110.0601, 124.0755, 138.0914, 152.1070, and 166.1228 ions for 5-OPA, 6-OHA, 7-OHA, 8-OOA, 9-ONA, and 10-ODA, respectively. 2,4-Decadienal (DDE)—another non-functionalized compound—also undergoes this process, yielding the C_10_H_16_N fragment ion at *m*/*z* 150.1279. However, the same process was observed for 4-hydroperoxynonenal (HPNE), leading to the C_9_H_16_O_2_N ion (*m*/*z* 170.1176), meaning that this cleavage may also occur at significant levels for different collision energies. Surprisingly, DDE was the only BBHA derivative to eliminate HBr from the MH^+^ precursor ion. Upon MS/MS, 4,5-epoxy-2-decenal (EDE) yielded two main fragment ions at *m*/*z* 254.0174 (C_11_H_13_NOBr) and *m*/*z* 201.9860 (C_7_H_9_NOBr), which could be attributed to cleavages at the epoxy function and the nitrogen atom, respectively.

As expected, the two dialdehydes—namely, malondialdehyde (MDA) and pyruvaldehyde (PRA)—were detected as their bis-BBHA derivatives, giving a characteristic isotopic pattern with ions at *m*/*z* 438.9651, 440.9631, and 442.9610, corresponding to their ^79^Br^79^Br, ^79^Br^81^Br, and ^81^Br^81^Br isotopologs, respectively. Despite their close chemical structure, these two derivatives yielded different fragment ions. Finally, it should be noted that all ω-oxo carboxylic acids except for 5-OPA gave a fragment ion at *m*/*z* 183.9756 fitting the molecular formula C_7_H_7_NBr, which appears to be specific to this series; its formation may also involve a complex rearrangement process, which was not investigated further.

Altogether, these MS/MS features show that although fragmentation processes are different depending on the structure of the BBHA derivatives, compounds belonging to the same class (e.g., HHE and HNE; OHE and ONE; ω-oxo carboxylic acids) show similar behavior towards MS/MS collisional activation, and that the corresponding fragment ions can be used for structural confirmation as well as for providing clues as to the class of compounds to which they belong. Furthermore, as previously mentioned, the *m*/*z* 168.9645 can be used for the tracking and confirmation of the BBHA derivatives using all-ion MS/MS strategies.

### 3.3. Profiling of Diet-Related Peroxidation Carbonyl Compounds Formed in the Intestinal Lumen

In order to assess the potential of the developed method for the untargeted profiling of lipid peroxidation aldehydes, fecal waters prepared from rats (*n* = 6) fed two distinct diets—namely, hydrogenated coconut oil (COHE) and linseed oil (LIHE)—containing different precursor fatty acid profiles, both with added heme iron, were derivatized with BBHA and analyzed via LC–HRMS. A post-analysis raw data-mining protocol was then set up to extract signals corresponding to brominated compounds (e.g., exact mass, isotopic pattern) coming from the derivatized aldehydes. Since many aldehydes produced by lipid oxidation are specific to their fatty acid precursors, several expected aldehyde derivatives (including, in particular, toxicologically relevant species such as hydroxy- and oxo-alkenals) were searched on the basis of the exact mass of their BBHA derivatives (i.e., both the ^79^Br and ^81^Br isotopic peaks) in a suspect screening approach. When a suspect signal was detected, confirmation was achieved using the standard BBHA derivatives synthesized in our previous works and used for the setup of the derivatization method. Signal intensities and fold changes between the two diets observed for the 16 compounds prepared as BBHA derivative standards in this suspect screening approach are reported in Supplementary Table S1. As expected, the occurrence of HHE was detected in fecal waters from rats fed the ω-3-fatty-acid-rich linseed oil, whereas it was not detected in fecal waters from rats fed hydrogenated coconut oil, mainly composed of saturated fatty acids. To a lesser extent, HNE and OHN were also detected from the linseed oil diet, which also contains ω-6 fatty acids, and not (OHN) or very weakly (HNE, fold change 10) detected in the coconut oil diet. In addition, linseed oil rich in α-linolenic acid gave important amounts of malondialdehyde (MDA, which was detected as its bis-BBHA derivative), whereas it was detected only in very weak amounts in the samples from rats fed coconut oil, leading to a fold change of 500. The other dialdehyde listed in Table 1—namely, PRA—was detected in similar amounts in both groups. This compound is known to result from carbohydrate fermentation, and is not considered to be a lipid peroxidation product [35]. Conversely, OHE, ONE, and EDE were not detected in any of the samples. Among the ω-oxo carboxylic acids, and as far as they can be considered to behave similarly towards electrospray ionization, 5-OPA and 9-ONA were the most abundantly detected (see Supplementary Table S1); although present in less abundance in the hydrogenated coconut oil diet, they were both detected in all samples. 9-ONA is known to be one of the main peroxidation products of ω-6 fatty acids [36], and its presence in all of the samples could be tentatively explained by basal lipid peroxidation of intestinal cells [37]. Finally, 8-OOA was the ω-oxo carboxylic acid displaying the highest fold change between the two diets. This observation illustrates the added value provided by untargeted approaches to get early warning messages to prioritize specific studies, which could be focused in this precise case on the origin of 8-OOA.

### 3.4. Untargeted “Aldehydomics” Approach: Statistical Data Analysis and Identification of Aldehydes

**Figure 3:**
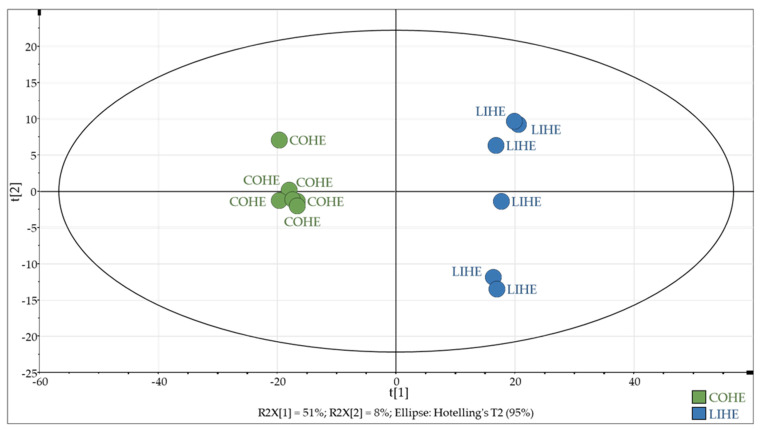
PCA performed on the 774 extracted BBHA-derivatized features. COHE: coconut oil supplemented with heme iron. LIHE: linseed oil supplemented with heme iron.

From the whole dataset acquired via full-scan HRMS from individual rat fecal waters obtained from the two diets differing in precursor fatty acid profiles, signals corresponding to brominated species were first extracted according to a mass measurement error of ± 5 ppm, using a homemade R script for filtering MS signals on the basis of (1) the mass difference between the ^79^Br and ^81^Br isotopes—i.e., 1.998 Da—and (2) the equivalent intensities of the two signals (see Materials and Methods). This process led to the extraction of 774 features containing only variables related to brominated compounds. This dataset was then subjected to multivariate statistical analysis using a classical global metabolomics method. As shown in Figure 3, the two diets were very well discriminated by principal component analysis (PCA), without the need for a supervised analysis, which confirms that BBHA-derivatized species are responsible for the observed separation.

After PLS-DA statistical analyses, 413 discriminating variables out of the 774 extracted features (corresponding to filtered brominated compounds) were validated with a VIP score > 1. A total of 84 features could be putatively annotated based on their exact mass using several databases (i.e., HMDB, LIPID MAPS, homemade database), among which 36 could be linked to lipids and their peroxidation products (the annotation table is reported in Supplementary Table S2). For several annotated compounds (namely, oxo-hexanoic acid, oxo-heptanoic acid, and oxo-eicosadienoic acid), standard compounds could be purchased and their corresponding BBHA derivatives synthesized. Unfortunately, the mass spectrometric features of the standard compounds did not fit those of the compounds detected in fecal waters. This enabled us to rule out only one of the possible isomeric structures for the annotated compounds, which remained at identification level 3 according to the Metabolomics Standards Initiative [38]. Finally, seven compounds were formally identified at identification level 1 thanks to the comparison with authentic standard compounds. These seven compounds are reported in Table 2.

As expected, MDA and HHE were identified among the variables exhibiting the highest VIP values (see Table 2). MDA was detected at *m*/*z* 438.9652 (Rt 18.6 min.) as its bis-BBHA derivative, whereas HHE was detected as its single-BBHA derivative at *m*/*z* 298.0437 (Rt 15.7 min.). Both compounds were confirmed at level 1 by comparison with the corresponding authentic standards. Although not representative of a single-precursor fatty acid, MDA represented the most significant annotated variable, and ranked third in the whole PLS-DA analysis. It displayed a fold change of 202, showing that the peroxidation rate of linseed-oil-fed rats’ feces was much higher than that of hydrogenated-coconut-oil-fed rats. With a fold change of 11, HHE was also among the most significant variables, confirming that it represents a major marker of ω-3 fatty acid peroxidation. HNE (*m*/*z* 340.0905, Rt 17.7 min.) was also identified at level 1 among the significant variables. The VIP and the fold change of this compound were not as elevated as for HHE, which was expected, since linseed oil is known to contain a majority of ω-3 fatty acids and a lesser proportion of ω-6 fatty acids. Surprisingly, some BBHA derivatives of fatty acids were also identified or annotated, although our previous trials in the derivatization buffer (without fecal water matrix) never produced any BBHA derivatives from fatty acid standards. Among them, eicosapentaenoic acid and α-linolenic acid were identified at level 1. It is noteworthy that in order to be able to make confirmation trials on these fatty acids, the derivatization process had to be carried out by spiking fecal waters, since no reaction occurs in the buffer medium used for the derivatization of standard compounds. One hypothesis for this observation may be found in the very high proportion of unchanged fatty acids coming from the oil-based diet (5% hydrogenated coconut or linseed oil in the diet) in the feces compared to lipid peroxidation products, which could explain the formation of their BBHA derivatives in a detectable abundance despite a very low reactivity. Another hypothesis may be found in the particular composition of fecal water that might have accelerated the reaction between BBHA and fatty acids, which are not prone to derivatization under “classical” chemical conditions. Note that a PCA statistical analysis carried out after removing these variables yielded the same result, showing that those unoxidized diet-sourced fatty acids were not influential for the discrimination of the two groups (data not shown).

**Figure 4:**
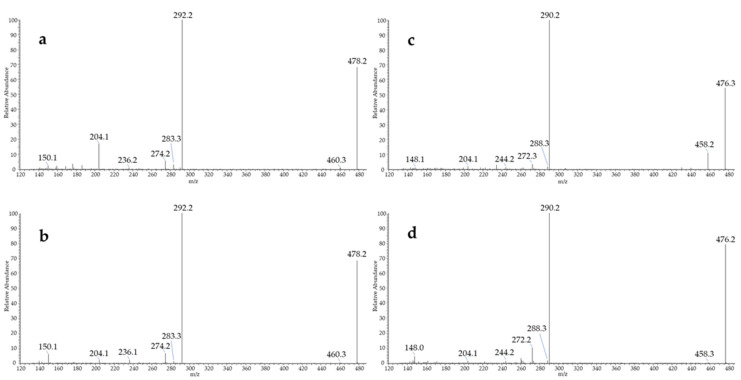
MS/MS spectra of the BBHA derivatives of 9-oxo-octadecadienoic acid obtained from (**a**) rat fecal water and (**b**) standard compound, and of the BBHA derivatives of 9-oxo-octadecatrienoic acid from (**c**) rat fecal water and (**d**) standard compound.

In addition to the expected MDA, HHE and, to a lesser extent, HNE, the developed method allowed for the identification of two additional lipid peroxidation compounds—namely, 9-oxo-octadecadienoic acid (9-oxo-ODE) and 9-oxo-octadecatrienoic acid (9-oxo-OTrE), which were not included in our first suspects. Their identity was confirmed at level 1 by comparison of their retention time, exact mass, and MS/MS spectra with those of the BBHA derivatives prepared from the authentic standard of the precursor oxo-fatty acids (Figure 4).

These oxo-fatty acids belong to the class of oxylipins—a family of compounds that come from the enzymatic or non-enzymatic oxidation of fatty acids, and have been reported to have biological activity [39]. In the case of two other compounds that could be annotated (see Supplementary Table S2) as oxo-eicosadienoic acid (*m*/*z* 506.2262) and oxo-octadecanoic acid (*m*/*z* 482.2206), the standard 15-oxo-eicosadienoic acid (*m*/*z* 506.2262) and 3-oxo-octadecanoic acid could be purchased for structural confirmation. Unfortunately, neither of these two compounds fit the retention time of our discriminating variables. This was also the case for the oxo-hexanoic acid and oxo-heptanoic acid, which could not be confirmed as the ω-oxo-acids by comparison with the BBHA standard derivatives, and for which an isomeric structure should also be considered.

## 4. Conclusions

The formation of lipid peroxidation products such as aldehydes is one of the most convincing hypotheses for linking increased CRC risk with red meat consumption. Aside from well-known reactive hydroxy-alkenals (e.g., HHE and HNE), several other lipid peroxidation products are likely to be formed, and a significant proportion of them are still unknown. Although metabolomics has become a powerful tool to identify biomarkers from various biological situations, it still suffers from a lack of capacity for the reliable annotation of several classes of metabolites. Moreover, in the case of lipid peroxidation products such as aldehydes, a specific approach has to be adopted in order to overcome the unstable character of these compounds, which prevents their analysis in their native form.

In this context, we developed an original analytical strategy based on the introduction of a bromine atom by derivatization. This allowed, on the one hand, the stabilization of aldehydes that are prone to degradation, and on the other hand, their selective detection after untargeted high-resolution mass spectrometry data acquisition, thanks to the selective filtration of the characteristic bromine isotopic pattern of derivatives, and therefore the selective filtration of aldehyde species. This allows access to semi-quantitative information, thereby comparing different situations by means of fold changes between signal intensities for a given compound. The developed method has been tested on fecal water samples from rats fed two contrasting diets according to their fatty acid composition. The results showed that the method allowed for (1) the detection of several expected aldehydes, including hydroxyl-alkenals such as HHE and HNE, by using a suspect screening approach, and (2) the detection of several other brominated signals, corresponding to potentially unknown aldehydes, among which several compounds could be identified in an untargeted manner. After this proof of concept developed on the basis of fecal water from rats fed with highly contrasting diets, we have shown that this method can be applied for the non-targeted detection of aldehydes for a much wider field of application. This opens the way for the easier identification of new carbonylated species resulting from lipid peroxidation.

## Figures and Tables

**Table 1:** List of prepared standard BBHA derivatives and their respective molecular mass, exact mass of the [M+H]^+^ ion, main MS/MS fragment ions, and chromatographic retention times.

Compound	Abbreviation	Chemical Formula BBHA Derivative	Molecular Mass	Calculated Exact Mass (^79^Br/^81^Br) or (^79^Br^79^Br/^79^Br^81^Br/^81^Br^81^Br) [M+H]^+^	Main Observed Fragment Ions (*m*/*z*) from MS/MS Carried Out on the ^79^Br Isotopomer	Retention Time (min.)
4-OH-hexenal	HHE	C_13_H_16_O_2_NBr	297/299	298.04372/300.04167	168.9645; 224.0069	15.6; 15.7
4-Oxo-hexenal	OHE	C_13_H_14_O_2_NBr	295/297	296.02807/298.02602	168.9645; 240.0020; 268.0329; 221.9912	16.8
4-OH-nonenal	HNE	C_16_H_22_O_2_NBr	339/341	340.09067/342.08862	168.9645; 224.0069	17.6; 17.8
4-Oxo-nonenal	ONE	C_16_H_20_O_2_NBr	337/339	338.07502/340.07297	168.9646; 240.0021; 221.9914; 310.0803	18.5
4,5-Epoxy-2-decenal	EDE	C_17_H_22_O_2_NBr	351/353	352.09067/354.08862	168.9645; 254.0174; 201.9860	17.8
2,4-Decadienal	DDE	C_17_H_22_ONBr	335/337	336.09575/338.09371	168.9649; 150.1279; 248.0072; 256,1698	20.3
4-Hydroperoxynonenal	HPNE	C_16_H_22_O_3_NBr	355/357	356.08558/358.08354	240.0022; 168.9648; 170.1176; 322.0807	17.7; 17.9
4-OH-nonanal	OHN	C_16_H_24_O_2_NBr	341/343	342.10632/344.10427	324.0960; 168.9642; 141.1273; 254,0175	17.6
Malondialdehyde	MDA	C_17_H_16_O_2_N_2_Br_2_ ^1^	438/440/442	438.96513/440.96308/442.96104	168.9645; 254.0048; 237.0021	18.7
Pyruvaldehyde	PRA	C_17_H_16_O_2_N_2_Br_2_ ^1^	438/440/442	438.96513/440.96308/442.96104	168.9650; 235.9948; 336.9224	19.8
5-Oxo-pentanoic acid	5-OPA	C_12_H_14_O_3_NBr	299/301	300.02298/302.02094	168.9649; 282.0128; 96.0044	14.5
6-Oxo-hexanoic acid	6-OHA	C_13_H_16_O_3_NBr	313/315	314.03863/316.03659	168.9649; 296.0286; 183.9758; 110.0601	15.0
7-Oxo-heptanoic acid	7-OHA	C_14_H_18_O_3_NBr	327/329	328.05428/330.05224	168.9648; 310.0441; 183.9761; 124.0755	15.5
8-Oxo-octanoic acid	8-OOA	C_15_H_20_O_3_NBr	341/343	342.06993/344.06789	168.9649; 324.0599; 183.9758; 138.0914	16.1
9-Oxo-nonanoic acid	9-ONA	C_16_H_22_O_3_NBr	355/357	356.08558/358.08354	168.9646; 338.0751; 183.9756; 152.1070	16.7
10-Oxo-decanoic acid	10-ODA	C_17_H_24_O_3_NBr	369/371	370.10123/372.09919	168.9648; 352.0910; 183.9758; 166.1228	17.2

^1^ bis-BBHA derivative.

**Table 2:** Lipid oxidation products and fatty acids identified at level 1 by the untargeted aldehydomics strategy.

Compound	*m*/*z* [M+H]^+^	Rt (min)	Identification Level	FoldLIHE/COHE
Malondialdehyde (MDA)	438.96519	18.60	1	202.27
4-Hydroxy-hex-2-enal (HHE)	298.04369	15.68	1	11.46
9-Oxo-octadecadienoic acid (9-oxoODE)	478.19490	20.51	1	18.49
9-Oxo-octadecatrienoic acid (9-oxoOTrE)	476.17916	19.93	1	78.39
4-Hydroxy-non-2-enal (HNE)	340.09047	17.65	1	3.27
α-Linolenic acid	462.20024	19.90	1	371.22
Eicosapentaenoic acid	486.19979	19.84	1	59.18

## Data Availability

The data presented in this study are available in article and supplementary material.

## Supplementary Materials

The following are available online at www.mdpi.com/article/10.3390/antiox10081261/s1, Table S1: List of screened carbonyl compounds, with their respective intensities in both diets, and fold changes between diets; Table S2: Annotation table of lipid peroxidation products from LC–HRMS analysis of rat fecal waters after BBHA derivatization.
