# Supplementary material for: Towards Aldehydomics: Untargeted Trapping and Analysis of Reactive Diet-Related Carbonyl Compounds Formed in the Intestinal Lumen
Source: Antioxidants (Basel). 2021 Aug 6;10(8):1261. doi: 10.3390/antiox10081261 (PMC8389236; doi:10.3390/antiox10081261)
Supplement: Supplementary file 1 [file antioxidants-10-01261-s001.zip › antioxidants-1277153-supplementary.pdf]

Table S1: List of screened carbonyl compounds with their respective intensities in both diets and fold changes between diets

Compound	Abbreviation	Chemical formula BBHA derivative	Molecular mass	Signal intensity COHE[1]	Signal intensity COHE[1]	Fold change LIHE / COHE
4-OH hexenal	HHE	$C_{13}H_{16}O_2NBr$	297 / 299	0	3.00E+05	NA[3]
4-oxo hexenal	OHE	$C_{13}H_{14}O_2NBr$	295 / 297	0	0	ND[2]
4-OH nonenal	HNE	$C_{16}H_{22}O_2NBr$	339 / 341	2.00E+04	2.00E+05	10
4-oxo nonenal	ONE	$C_{16}H_{20}O_2NBr$	337 / 339	0	0	ND[2]
4,5-epoxy-2-decenal	EDE	$C_{17}H_{22}O_2NBr$	351 / 353	0	0	ND[2]
2,4-decadienal	DDE	$C_{17}H_{22}ONBr$	335 / 337	2.00E+04	2.00E+04	1
4-hydroperoxynonenal	HPNE	$C_{16}H_{22}O_3NBr$	355 / 357	3.00E+04	5.00E+04	1.67
4-OH nonanal	OHN	$C_{16}H_{24}O_2NBr$	341 / 343	0	1.00E+05	NA[3]
Malondialdehyde	MDA	$C_{17}H_{16}O_2N_2Br_2$	438 / 440 / 442	2.00E+04	1.00E+07	500
Pyruvaldehyde	PRA	$C_{17}H_{16}O_2N_2Br_2$	438 / 440 / 442	3.00E+05	3.00E+05	1
5-oxo-pentanoic acid	5-OPA	$C_{12}H_{14}O_3NBr$	299 / 301	3.00E+06	3.00E+06	1
6-oxo-hexanoic acid	6-OHA	$C_{13}H_{16}O_3NBr$	313 / 315	3.00E+05	4.00E+05	1.33
7-oxo-heptanoic acid	7-OHA	$C_{14}H_{18}O_3NBr$	327 / 329	3.00E+05	4.00E+05	1.33
8-oxo-octanoic acid	8-OOA	$C_{15}H_{20}O_3NBr$	341 / 343	1.00E+05	1.00E+06	10
9-oxo-nonanoic acid	9-ONA	$C_{16}H_{22}O_3NBr$	355 / 357	2.00E+06	4.00E+06	2
10-oxo-decanoic acid	10-ODA	$C_{17}H_{24}O_3NBr$	369 / 371	1.00E+05	4.00E+05	4

[1] Arbitrary Unit

[2] ND: not detected

[3] NA: not applicable (dividing by zero)

Table S2: Annotation table of lipid peroxidation products from LC-HRMS analysis of rat fecal waters after BBHA derivatization.

Proposed compound	m/z [M+H]+	Parent compound mass	Parent compound MF	Rt (min)	Identification level	nb BBHA	Fold LIHE/COHE	Origin and references
Dialdehyde/diketone								
Malondialdehyde	438.96519	72.02113	$C_3H_4O_2$	18.60	1	2	202.27	Oxidation of PUFAs with more than two methylene-interrupted double bonds [1]
Pentanedione	466.99611	100.05205	$C_5H_8O_2$	21.35	3	2	3.52	Lipid oxidation product [2]
Alkanal/alkenal								
Hexanal	284.06444	100.08904	$C_6H_{12}O$	18.89	3	1	2.19	Oxidation of linoleic acid [1]
2-Heptenal	296.06442	112.08901	$C_7H_{12}O$	19.05	3	1	2.17	Oxidation of various oils [2]
Hydroxy-aldehyde								
Lactaldehyde	258.01239	74.03698	$C_3H_6O_2$	12.68	3	1	12.41	Methyl-glyoxal metabolite
Hydroxy-alkenal and metabolites								
4-Hydroxy-hexenal	298.04369	114.06829	$C_6H_{10}O_2$	15.68	1	1	11.46	Oxidation of n-3 PUFAs [1]
4-Hydroxy-octenal	326.07485	142.09944	$C_8H_{14}O_2$	16.39	3	1	5.13	Oxidation of linoleic acid [1]
4-Hhydroxy-nonenal	340.09047	156.11507	$C_9H_{16}O_2$	17.65	1	1	3.27	Oxidation of n-6 PUFAs [1]
9-Carboxy-4-hydroxy-nonanal	372.08038	188.10498	$C_9H_{16}O_4$	13.64	3	1	89.71	HNE metabolite [3]
10-Carboxy-4-hydroxy-decenal	384.08052	200.10512	$C_{10}H_{16}O_4$	14.39	3	1	135.5	4-Hydroxy-decenal metabolite (on basis of HNE biotransformation pathways)
10-Carboxy-4-oxo-decenoic acid	398.05986	214.08450	$C_{10}H_{14}O_5$	14.24	3	1	13.67	4-Oxo-decenal metabolite
10-Carboxy-deca-2,4-dienal	366.06969	182.09429	$C_{10}H_{14}O_3$	16.70	3	1	63.63	2,4-Decadienal metabolite-linoleic acid oxidation product [4]
4-Hydroxy-pentadecenal	424.18437	240.20897	$C_{15}H_{28}O_2$	19.88	3	1	2.42	Lipid oxidation product [5]
Di-hydroxy-alkenals and metabolites								
8-Carboxy-4,5-dihydroxy-octenal	372.04414	188.06874	$C_8H_{12}O_5$	13.63	3	1	1.58	4,5-Dihydroxy-octenal metabolite (on basis of HNE biotransformation pathways)
4,5-Dihydroxy-decenal	370.10097	186.12557	$C_{10}H_{18}O_3$	18.44	3	1	5.59	Oxidation of n-6 PUFAs [1]
10-Oxo-4,5-dihydroxydecenal	567.04907	200.10501	$C_{10}H_{16}O_4$	19.71	3	2	12.7	4,5-Dihydroxy-decenal metabolite (on basis of HNE biotransformation pathways)
10-Carboxy-4,5-dihydroxy-decenal	400.07545	216.10005	$C_{10}H_{16}O_5$	12.96	3	1	55.59	4,5-Dihydroxy-decenal metabolite (on basis of HNE biotransformation pathways)
11-Carboxy-4,5-dihydroxy-undecenal	414.09112	230.11572	$C_{11}H_{18}O_5$	14.83	3	1	1.47	4,5-Dihydroxy-undecenal metabolite (on basis of HNE biotransformation pathways)
12-Carboxy-4,5-dihydroxy-dodecenal	428.10671	244.13131	$C_{12}H_{20}O_5$	15.31	3	1	7.89	4,5-Dihydroxy-dodecenal metabolite (on basis of HNE biotransformation pathways)
14-Carboxy-4,5-dihydroxy-tetradecenal	456.13789	272.16249	$C_{14}H_{24}O_5$	17.45	3	1	11.55	4,5-Dihydroxy-tetradecenal metabolite (on basis of HNE biotransformation pathways)
Oxo-fatty acids								

Dioxo-dodecenoic acid	593.06435	226.12029	$C_{12}H_{18}O_4$	19.16	3	2	14.13	Di-oxo fatty acid, product of non enzymatic oxidation of linoleic acid [6]
Oxo-pentadecadienoic acid	450.16356	266.18816	$C_{16}H_{26}O_3$	19.55	3	1	8.45	Oxo-fatty acid
Oxo-octadecanoic acid	482.22605	298.25065	$C_{18}H_{34}O_3$	18.60	3	1	12.71	Oxo-fatty acid
Hydroxy-oxo-octadecenoic acid	496.20468	312.22928	$C_{18}H_{32}O_4$	18.20	3	1	5.01	Hydroxy-oxo-fatty acid, product of non enzymatic oxidation of linoleic acid [6]
9-Oxo-octadecadienoic acid	478.19490	294.21950	$C_{18}H_{30}O_3$	20.51	1	1	18.49	Oxo-fatty acid [7,8]
9-Oxo-octadecatrienoic acid	476.17916	292.20376	$C_{18}H_{28}O_3$	19.93	1	1	78.39	Oxo-fatty acid [7]
Epoxy-oxo-octadecenoic acid or oxo-hydroxy-octadecatrienoic acid	494.18942	310.21402	$C_{18}H_{30}O_4$	19.33	3	1	5.12	Epoxy-oxo derivative of linoleic acid [8] or oxo-hydroxy derivative of linolenic acid [9]
Oxo-nonadecanoic acid	496.24186	312.26646	$C_{19}H_{36}O_3$	24.21	3	1	2.97	Oxo-fatty acid
Oxo-eicosadienoic acid	506.22621	322.25081	$C_{20}H_{34}O_3$	23.24	3	1	46.1	Oxo-fatty acid (not 15-oxo-eicosa-11Z,13E-dienoic acid)
Oxo-eicosanoic acid	510.25736	326.28196	$C_{20}H_{38}O_3$	25.37	3	1	3.95	Oxo-fatty acid
Others								
Eicosapentaenoic acid	486.19979	302.22439	$C_{20}H_{30}O_2$	19.84	1	1	59.18	Fatty acid
◎-linolenic acid	462.20024	278.22484	$C_{18}H_{30}O_2$	19.90	1	1	371.22	Fatty acid
11-Deoxyprostaglandin E1	522.22093	338.24553	$C_{20}H_{34}O_4$	21.33	3	1	62.1	Prostaglandin
5-Oxo-2-amino-valeric acid	315.03374	131.05834	$C_5H_9NO_3$	11.24	3	1	4.59	Oxidation product of arginine [10]
Oxo-hexanoic acid	314.03857	130.06317	$C_6H_{10}O_3$	15.87	3	1	0.46	Oxo-acid (not 6-oxo-hexanoic acid)
Oxo-heptanoic acid	328.05408	144.07868	$C_7H_{12}O_3$	13.78	3	1	0.38	Oxo-acid (not 7-oxo-heptanoic acid)

References

1. Esterbauer, H.; Schaur, R.J.; Zollner, H. Chemistry and Biochemistry of 4-Hydroxynonenal, Malonaldehyde and Related Aldehydes. *Free Radic. Biol. Med.* **1991**, *11*, 81–128.

2. Nieva-Echevarría, B.; Goicoechea, E.; Guillén, M.D. Polyunsaturated Lipids and Vitamin A Oxidation during Cod Liver Oil in Vitro Gastrointestinal Digestion. Antioxidant Effect of Added BHT. *Food Chem* **2017**, *232*, 733–743, doi:10.1016/j.foodchem.2017.04.057.

3. Keller, J.; Baradat, M.; Jouanin, I.; Debrauwer, L.; Guéraud, F. “Twin Peaks”: Searching for 4-Hydroxynonenal Urinary Metabolites after Oral Administration in Rats. *Redox Biol* **2015**, *4*, 136–148, doi:10.1016/j.redox.2014.12.016.

4. Spiteller, P.; Kern, W.; Reiner, J.; Spiteller, G. Aldehydic Lipid Peroxidation Products Derived from Linoleic Acid. *Biochim Biophys Acta* **2001**, *1531*, 188–208, doi:10.1016/s1388-1981(01)00100-7.

5. Curzio, M.; Esterbauer, H.; Di Mauro, C.; Cecchini, G.; Dianzani, M.U. Chemotactic Activity of the Lipid Peroxidation Product 4-Hydroxynonenal and Homologous Hydroxyalkenals. *Biol Chem Hoppe Seyler* **1986**, *367*, 321–329, doi:10.1515/bchm3.1986.367.1.321.

6. Zhu, X.; Tang, X.; Anderson, V.E.; Sayre, L.M. Mass Spectrometric Characterization of Protein Modification by the Products of Nonenzymatic Oxidation of Linoleic Acid. *Chem Res Toxicol* **2009**, *22*, 1386–1397, doi:10.1021/tx9000072.

7. Gabbs, M.; Leng, S.; Devassy, J.G.; Monirujjaman, M.; Aukema, H.M. Advances in Our Understanding of Oxylipins Derived from Dietary PUFAs. *Adv Nutr* **2015**, *6*, 513–540, doi:10.3945/an.114.007732.

8. Vangaveti, V.N.; Jansen, H.; Kennedy, R.L.; Malabu, U.H. Hydroxyoctadecadienoic Acids: Oxidised Derivatives of Linoleic Acid and Their Role in Inflammation Associated with Metabolic Syndrome and Cancer. *Eur J Pharmacol* **2016**, *785*, 70–76, doi:10.1016/j.ejphar.2015.03.096.

9. Davoine, C.; Douki, T.; Iacazio, G.; Montillet, J.-L.; Triantaphylidès, C. Conjugation of Keto Fatty Acids to Glutathione in Plant Tissues. Characterization and Quantification by HPLC-Tandem Mass Spectrometry. *Anal Chem* **2005**, *77*, 7366–7372, doi:10.1021/ac051155y.

10. Spiteller, G. Linoleic Acid Peroxidation--the Dominant Lipid Peroxidation Process in Low Density Lipoprotein--and Its Relationship to Chronic Diseases. *Chem Phys Lipids* **1998**, *95*, 105–162, doi:10.1016/s0009-3084(98)00091-7.
